# The relationship between serum vitamin D and fracture risk in the elderly: a meta-analysis

**DOI:** 10.1186/s13018-020-01603-y

**Published:** 2020-02-27

**Authors:** Ning Wang, Yungang Chen, Jindou Ji, Jinlei Chang, Shengwen Yu, Bo Yu

**Affiliations:** 1 Shandong University of Traditional Chinese Medicine, Jingshi Road16369, Jinan, 250014 China; 2 Department of Orthopaedics, Affiliated Hospital of Shandong University of Traditional Chinese Medicine, Jingshi Road16369, Jinan, 250014 China

**Keywords:** Serum 25-hydroxyvitamin D, Osteoporotic fracture, Hip fracture, Meta-analysis

## Abstract

**Background:**

The incidence of osteoporotic fractures has increased rapidly, and because of the poor prognosis and high mortality associated with osteoporotic fractures, they remain a prospective research area globally. One way to reduce their incidence is to investigate their intervention risk factors in the elderly. Hence, this study explores the correlation between serum 25-hydroxyvitamin D [25(OH)D] levels and osteoporotic fractures in elderly patients through a meta-analysis.

**Methods:**

We conducted our literature search mainly in PubMed and Embase for identifying studies that investigated the relationship between serum 25(OH)D levels and the risk for osteoporotic fractures. We performed categorical analysis, heterogeneity checks, publication bias analysis, and subgroup analyses.

**Results:**

In total, 20 studies were included, of which 4 were case-cohort studies and 16 were cohort studies. A total of 41,738 patients from 20 studies were included in the meta-analysis, of which 5916 had fractures, including 3237 hip fractures. By combining the lowest and highest categories of relative risks (RRs) and 95% confidence intervals (CIs), it was suggested that lower serum 25-hydroxyvitamin D levels may be a risk factor for fractures. RR (95% CI) for total and hip fractures were 1.11 (0.99, 1.24) and 0.89 (0.80, 0.98) after adjustments.

**Conclusions:**

Our study showed that compared to low serum 25(OH)D levels, high serum 25(OH)D levels reduce the risk of hip fractures in the patients aged 60 years or older. In contrast, serum 25(OH)D has no significant relationship with total fracture risk.

## Background

As the growth of population worldwide, the incidence of osteoporotic fractures is rising rapidly as well [1]. The lifetime risk of osteoporotic fractures remains high, accounting for 40–50% of women and 13–22% of men in western countries. When elderly patients (patients aged 65 and above) are affected by osteoporotic fractures, they have to be hospitalized, given long-term care, experience a decline in the quality of life, or may have adverse outcomes such as disability or death [2]. Therefore, osteoporosis and osteoporotic fractures remain a serious public health problem globally. Exploring the interventional risk factors for osteoporotic fractures in the elderly such as BMI (body mass index) and PA (physical activity) can be instrumental in understanding the disease better. Even serum markers, including magnesium, copper, iron, and vitamins, can be considered as new risk factors associated with the incidence of osteoporotic fracture in the elderly.

Serum 25-hydroxyvitamin D [25(OH)D] is recognized as the main circulating form of vitamin D, which accurately indicates the vitamin D concentration in the body. It reflects the nutritional status of vitamin D. Bone mineral density (BMD), bone size (relative to body size), and the bone strength impacted by the level of serum 25(OH)D [3, 4]. Previous studies show a correlation between low serum vitamin D and the risk of osteoporotic fractures. However, these findings are debatable [5–8]. Looker et al. [9] indicated that low serum vitamin D is associated with the occurrence of osteoporotic hip fractures in the elderly. Similar conclusions were obtained in the study by Holvik et al. [10]. However, Ginsberg et al. [11] showed that there is no association between serum 25(OH)D and hip fractures, and that by Barbour et al. [12] showed that serum 25(OH)D is unrelated with the occurrence of any non-spine fractures. In a 2017 meta-analysis on the relationship between serum 25(OH)D and the risk of total fractures, it was found that serum 25(OH)D is inversely proportionate to the incidence of total fractures [13].

However, it is worth noting that due to the different bone turnover rates, osteoporotic fractures in the elderly are of two types: perimenopausal and senile osteoporotic fractures [14]. When these two types are evaluated together, it becomes inconclusive.

There is no meta-analysis that presents the association between serum 25(OH)D and the risk for osteoporotic fractures in the elderly older than 60 years. Our study answers the following question for serum 25(OH)D and osteoporotic fractures: What is the relationship between serum 25(OH)D and hip and total fractures in elderly older than 60 years?

## Methods

We performed a meta-analysis of the available literature according to the PRISMA statement guidelines [15]. Ethical approval and written informed consent from patients were not necessary because our study was based on summaries and analyses of results of existing studies.

### Search strategy and data sources

We searched PubMed and EMBASE systematically using the following keywords: (1) “serum 25-hydroxyvitamin D,” “serum 25(OH) D, OR serum vitamin D;” (2) “hip fractures OR “total fractures OR fractures;” (3) “cohort study OR case-cohort studies OR prospective study OR prospective studies” The search time is up to December 2019, and there are no restrictions on language and year of publication. To avoid initial misses, we scanned the related articles and used the “related article” function for extra searches. The full text of all citations that appeared relevant was inspected by two independent reviewers. Furthermore, we also manually searched the abstract of meetings related to Endocrinology and Traumatology, which provided printed or electronic publications. However, we did not cite these meeting abstracts in this study.

### Study selection

All included studies were independently assessed by two reviewers. If there is any objection about data inclusion and data interpretation, it is resolved through arbitration and an agreement is reached after discussion.

Characteristics of included studies are as follows: (1) observational study, (2) the patients had hip fractures or some other type of fractures and reported the corresponding serum 25(OH)D levels, or (3) calculated and reported the value of relative risks (RR), odds ratios (OR), or hazard ratios (HR) and 95% confidence interval (CIs). (4) The study population was aged ≥ 60.

The exclusion criteria were as follows: (1) retrospective studies; (2) persons aged below 60 years; (3) serum 25(OH)D levels and hip or total fractures, RR, OR, or HR and 95% CI were not stated; (4) the full conference abstract was not found; and (5) the study population has a disability or other disease that affects the final outcome.

### Data extraction

Two reviewers independently extracted the data using a standardized data collection form. Discrepancies were resolved through discussion with other investigators and through reference to the original articles. The following data were extracted from each study: the first author’s last name, year of publication, type of study, country of study, gender and age of participants, years of follow-up, sample size, number of fractures, threshold of 25(OH)D levels, adjusted variables, and corresponding 95% CIs-RR estimates; if RR of different potential confounding factors is higher, the RR we extracted reflects the maximum control of potential confounding factors. If required, we contacted the authors of the preliminary study for more information.

### Statistical analyses

Relative risks (RR) were required as the general measure of association across studies. HRs and ORs were transformed into RRs [16–18].

For the meta-analysis, we performed a random-effects model [19]. Cochran Q statistics and *I*^2^ statistics were used to evaluate heterogeneity between studies [20]. We followed the suggestions of Higgins et al.; *I*^2^ values of 25%, 50%, and 75% were considered low, moderate, and high, respectively [21]. For *P* < 0.10 values of the Cochran Q statistic, we considered statistical heterogeneity and we report a random-effects model. Subgroup analyses were conducted to assess associations between the fracture risk and relevant study characteristics (gender, region, the starting time of the study) as possible sources of heterogeneity. Subgroup analysis was used for classified variables. We used funnel plot asymmetry to examine publication bias, and the Egger regression test was used to measure the asymmetry of the funnel plot [22]. We conducted the “trim and fill” assessment to further assess the possible effect of publication bias in our meta-analysis additionally. This method can reflect the positive studies that cause funnel plot asymmetry by conservatively imputing hypothetical negative unpublished studies [23, 24]. All statistical analyses were conducted using Stata 12 (StataCorp, College Station, Texas).

## Result

### Search results

**Fig. 1:**
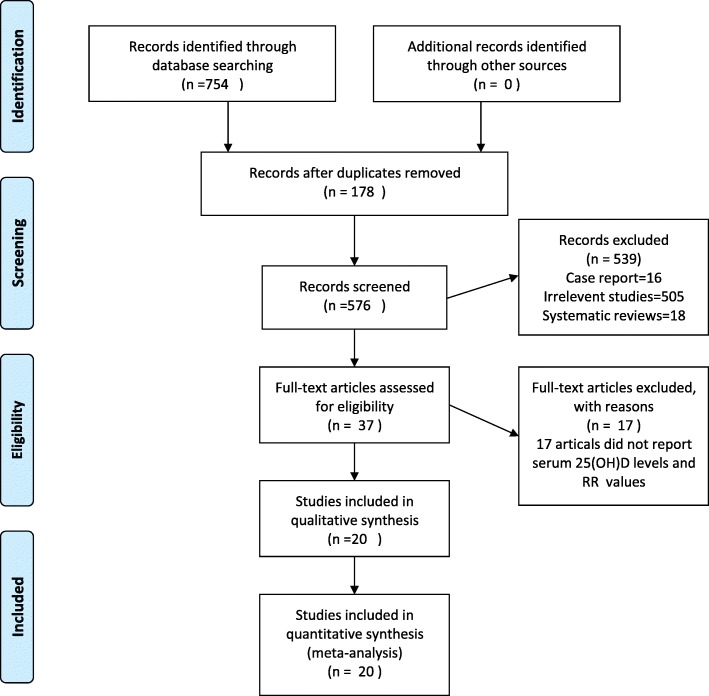
Flowchart of the study selection

The PRISMA statement flowchart shows the process of literature screening, study selection, and reasons for exclusion (Fig. 1). The initial database search included a total of 754 studies. After reading abstracts and titles, 717 studies were excluded, including 178 duplicate articles and 539 articles that did not meet the inclusion criteria. We then assessed the quality of the remaining 37 articles and excluded 17 of them. Finally, we selected 20 articles for this meta-analysis [5–7, 9–12, 25–37].

### Study characteristics

In total, 41,738 patients from 20 studies were obtained in the meta-analysis and 5916 had fractures, including 3237 hip fractures. The characteristics of the studies and participants are summarized in Table 1. The timeline of the studies was as follows: 4 studies were conducted before 2010, and 16 articles were written after 2010. Of the 20 studies, there were 16 cohort studies and 4 case-cohort studies; three of them were conducted in Asia, six studies were conducted in Europe, two were performed in Australia, and nine proceeded in the USA. The risk estimates provided by the vast majority of studies have been adjusted for age, sex, drinking, smoking, BMI, physical activity, and weight. Four articles included men only, 6 studies involved women, and the other 10 studies were conducted including men and women. Patients participated together; the longest follow-up time was 13.1 years and the shortest was 4 years.

### Serum 25(OH)D level and total fracture

**Fig. 2:**
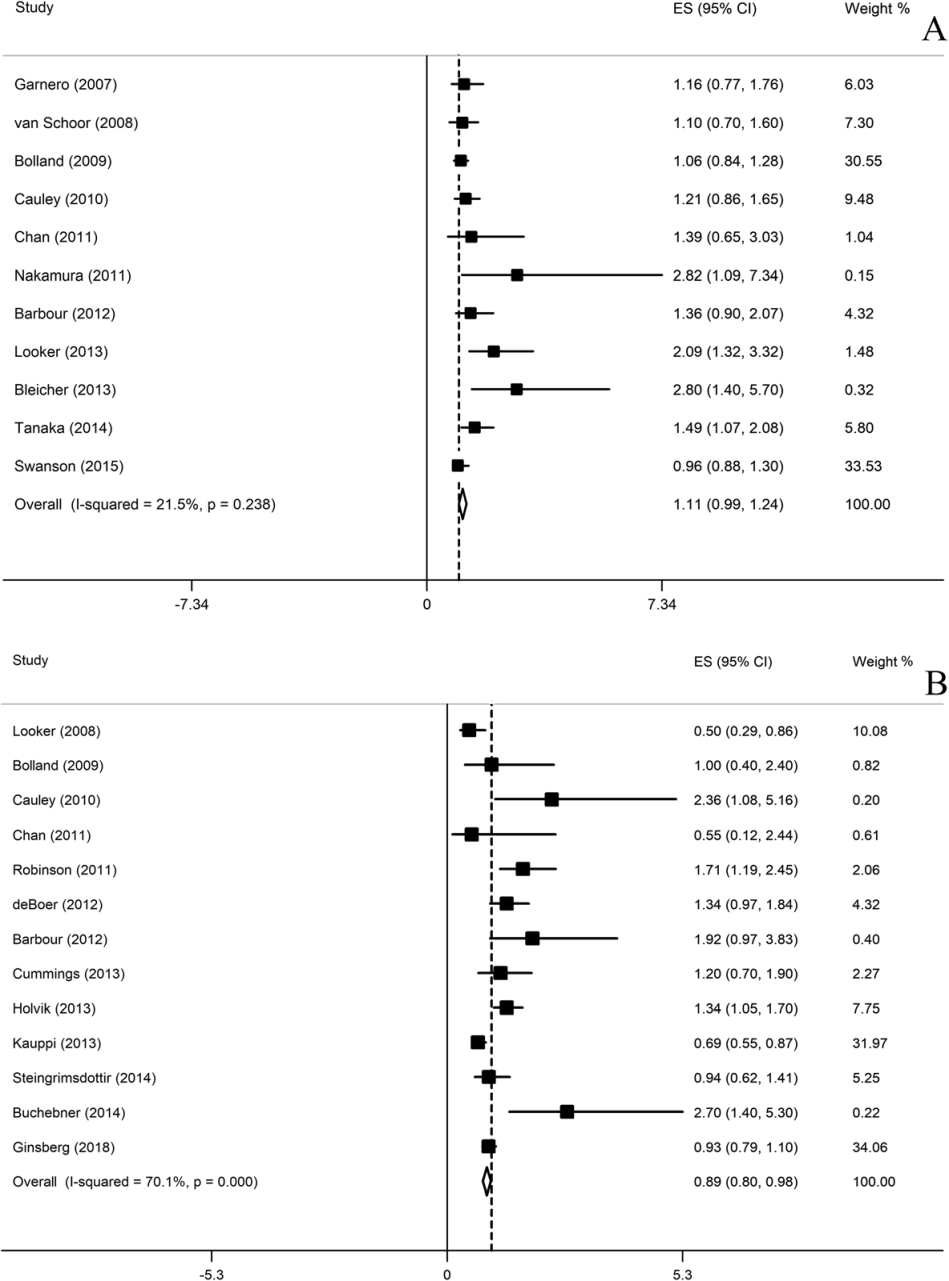
Adjusted relative risk (RR) of 25-hydroxyvitamin D (serum 25(OH) vitamin D) level and total fracture (**a**) and hip fracture (**b**) risk

**Fig. 3:**
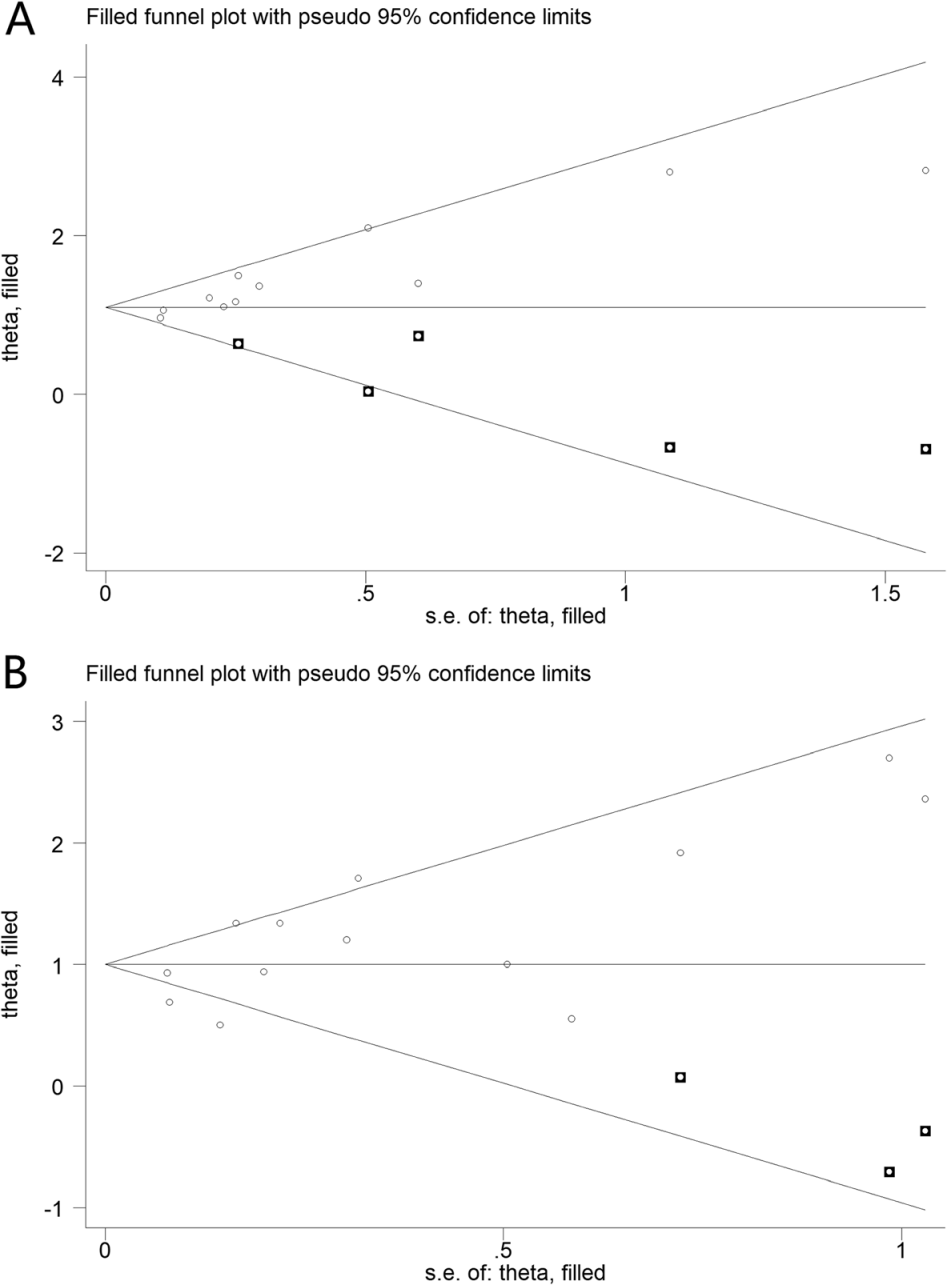
Trim and fill funnel plot for meta-analysis of the association between 25(OH)D level and total and hip fracture

A total of 21,837 participants were included and 2986 fracture events were obtained in this analysis. All the 11 studies were prospective cohort studies on fracture analysis. Figure 2a shows the results of the meta-analysis. The multivariable-adjusted relative risks (95% CI) of serum 25(OH)D level was 1.11 (0.99, 1.24). There was low heterogeneity across studies (*P* = 0.238; *I*^2^ = 21.5%). The pooled estimate of serum 25(OH)D level and the risk of hip fracture events did not vary substantially with the exclusion of anyone study by sensitivity analysis (Fig. 3a).

### Serum 25(OH)D level and hip fracture

A total of 24,220 participants were included and more than 2831 hip fracture events were obtained in this analysis. All 13 studies were prospective cohort studies on hip fracture analysis. Figure 2b shows the results of the meta-analysis. The multivariable-adjusted relative risks (95% CI) of serum 25(OH)D level was 0.89 (0.80, 0.98). There was high heterogeneity across studies (*P* = 0.000; *I*^2^ = 70.1%). The pooled estimate of serum 25(OH)D level and the risk of hip fracture events did not vary substantially with the exclusion of anyone study by sensitivity analysis (Fig. 3b).

### Publication bias

**Fig. 4:**
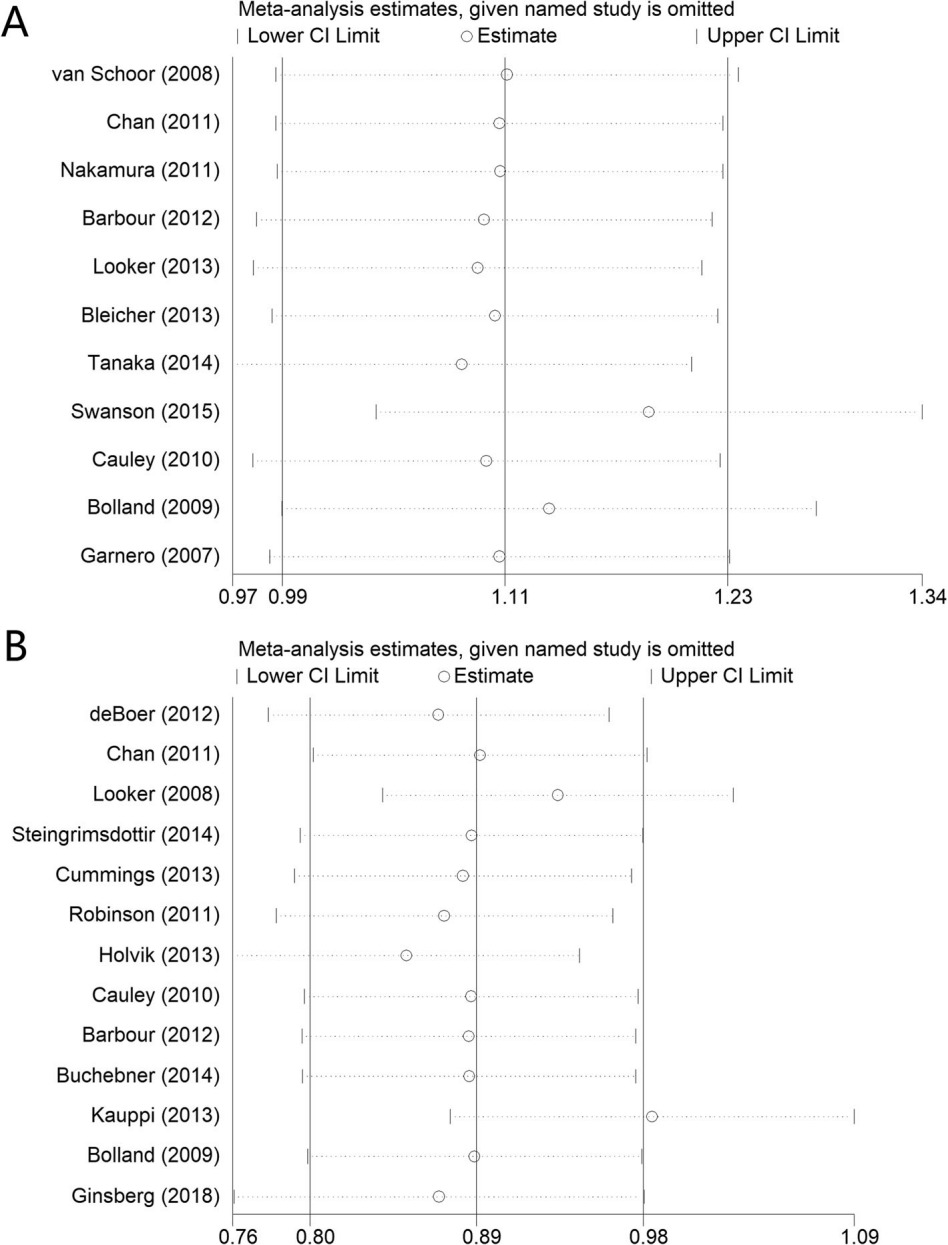
Sensitivity analysis of serum 25-hydroxyvitamin D (25(OH) vitamin D) level and total fracture (**a**) and hip fracture (**b**) risk

For total fracture, Begg’s test (*P* = 0.01) shows the possibility of publication bias. We conducted the trim and fill analysis additionally (Fig. 4a). This method can indicate the positive studies that cause funnel plot asymmetry by conservatively imputing hypothetical negative unpublished studies. The adjusted summary RR was based on the final result of the filled funnel plot (1.09, 95% CI 0.91, 1.28, *P* < 0.001), which did not vary substantially.

For hip fracture, Begg’s test (*P* = 0.39) indicates that in the analysis by fracture site, there was no publication bias between serum vitamin D levels and hip fracture events. The trim and fill analysis show the similar results (Fig. 4b).

### Subgroup analysis

A subgroup analysis of the relativity between serum 25 (OH)D levels and the risk of fracture was conducted. The results are shown in Table 2. We tested the possibility of gender as a source of heterogeneity. Ten studies included single-sex participants. For hip fracture, the relative risks (95% CL) were 0.99 (− 0.02 to 2.00) for males and 1.25 (0.75 to 1.75) for females, and for total fracture, the relative risks (95% CL) were 1.04 (0.85 to 1.22) for males and 1.14 (0.95 to 1.33) for females. We also tested region as a possible source of heterogeneity. For all studies, the relative risks (95% CL) were 0.88 (0.76 to 1.01) for studies proceeded in Europe; 1.50 (1.04 to 1.96) for studies proceeded in Asia; 0.98 (0.87 to 1.08) for studies proceeded in the USA, and 1.08 (0.86 to 1.30) for studies proceeded in Australia. We also performed a subgroup analysis based on the starting time of the studies to test the sources of heterogeneity. For hip fracture, the RR (95% CI) was 1.11 (0.93 to 1.28) for the studies started before the year 2000 and 1.12 (0.95 to 1.29) for the studies started after the year 2000. For total fracture, it is 0.99 (0.87 to 1.10) for the studies started before the year of 2000 and 0.73 (0.58 to 0.88) for the studies started after the year 2000.

**Table 1:** Summary characteristics of studies and participants

Name	Year	Study type	TF/HF/N	Sex	Age	Country	Period	Follow-up (year)	Adjustment
van Schoor et al	2008	Cohort	115\0\1311	Both	≥ 65	Netherland	1992–1993	6	Age, sex, season, education, BMI, number of chronic, diseases, creatinine level, cognition, smoking, and alcohol use
Chan et al	2011	Cohort	72\24\712	Men	≥ 65	China	2001–2003	4	Age, BMI, education, PASE, DQI, smoking status, and alcohol use
Nakamura et al	2011	Cohort	51\0\4250	Women	≥ 69	Japan	2009	6	Age, BMI, BMD, medication of osteoporosis and PA
Barbour et al	2012	Cohort	331\84\2614	Both	≥ 70	USA	1997–1998	6.4	Age, gender, race, education level, season of blood draw, BMI, current drinking, fracture after age 45 and clinical comorbidity index
Looker et al	2013	Cohort	525\287\4749	Both	≥ 65	USA	2000–2004	7	Age, sex, race/ethnicity, and survey
Bleicher et al	2013	Cohort	123\0\1662	Men	70–79	Australia	2005–2007	4.3	Age, country of birth, BMI, PA, season of blood draw, previous fracture after age 50 years, calcium and vitamin D supplement, and BMD
Tanaka et al	2014	Cohort	382\42\1470	Women	63.7 ± 10.7	Japan	2003	7.2	BMD, age, weight, diabetes mellitus, PTH, eGFR, prior fracture, back pain, bisphosphonates, SERM, active vitamin D
Swanson et al	2015	Case cohort	432\81\1000	Men	74.6 ± 6.2	USA	2000–2002	4.5	Age, race, site, season, PA, height, weight, baseline hip BMD,1,25(OH)2D measure, and incident falls in the first year of follow-up
Cauley et al	2010	Case cohort	436\81\1929	Men	≥ 65	USA	2000–2002	5.3	Age, race, clinic, season of blood draw, physical activity, weight, and height
Bolland et al	2009	Cohort	385\22\1471	Women	Mean 74	New Zealand	1998–2003	5	Treatment allocation (calcium or placebo) and baseline age, body weight, and smoking status
Garnero et al	2007	Cohort	134\0\669	Women	Mean62.2	France	1992–1993	11.2	Age, prevalent fracture, and PA
deBoer et al	2012	Cohort	0\137\1621	Both	≥ 65	USA	1992–2006	11	Age, sex, clinical site, smoking, body mass index, and physical activity.
Looker et al	2008	Cohort	0\156\1917	Both	≥ 65	USA	1988–1994	6.7	Age, sex, femoral neck BMD, BMI, previous fracture, dietary calcium, kilocalories, and weight loss from maximum
Steingrimsdottir et al	2014	Cohort	0\261\5471	Both	≥ 66	Iceland	2002–2006	5.4	Age, sex, body mass index, height, smoking, alcohol intake and season, physical activity
Cummings et al	2013	Cohort	271\133\878	Women	≥ 65	USA	1986–1988	5.9	Age and weight
Robinson et al	2011	Cohort	0\242\2249	Both	≥ 65	USA	1989–2009	13	Age, race, sex, clinic site, a season, education, smoking status (never smoker, former smoker, or current, smoker), alcohol use (any vs. none), diabetes status (normal, impaired fasting glucose, or diabetes), body mass index, self-reported health status, physical activity level, oral steroid use, estrogen use, thiamine and loop diuretic use, serum cystatin C level, and calcium supplement use
Holvik et al	2013	Case cohort	0\1175\2526	Both	≥ 65	Norway	1994–2001	10.7	Age, gender, study center, BMI, and month of blood sample
Buchebner et al	2014	Cohort	349\130\1044	Women	75	Sweden	1995–1999	13.1	smoking, bisphosphonate use, and physical activity level
Kauppi et al	2013	Cohort	0\95\3305	Both	62.9	Finland	2000–2001	8.4	Gender, age, height, weight, BMI, QU, alcohol consumption, smoking, and PA
Ginsberg et al	2018	Case cohort	0\289\890	Both	Mean78	USA	1989–2006	8.4	Age, sex, race, season of measurements, site of measurement and BMIeGFR, serum calcium, phosphate and FGF-23

*TF* total fractures, *HF* hip fractures, *N* cases

**Table 2:** Subgroup analysis to investigate differences between studies included in meta-analysis

Subgroup	Studies	RR (95% CI)	*I*^2^ (%)	*P* value
Gender				
TF				
Men	4	1.04 (0.85, 1.22)	28.1	0.24
Women	4	1.14 (0.95, 1.24)	13.5	0.33
HF				
Men	2	0.99(−0.02, 2.00)	56.2	0.13
Women	3	1.25 (0.75, 1.75)	16.3	0.3
Region				
Europe	6	0.88 (0.76, 1.01)	73.3	0
Asia	3	1.5 (1.04, 1.96)	0	0.7
USA	9	0.98 (0.88, 1.08)	70.7	0
Australia	2	1.08 (0.86, 1.30)	59.8	0.12
Start time				
TF				
Before 2010	4	1.11 (0.93, 1.28)	0	0.82
After 2010	7	1.12 (0.99, 1.24)	49.1	0.07
HF				
Before 2010	8	0.99 (0.87, 1.10)	72.4	0
After 2010	4	0.73 (0.58, 0.88)	22.5	0.28

*TF* total fractures, *HF* hip fractures

## Discussion

This meta-analysis was based on a total of 41,738 participants and 5916 patients (including 3237 hip fractures) who were older than 60 years indicated that serum 25 hydroxyvitamin D levels were negatively correlated to the risk of hip fracture. However, serum 25(OH)D has no association with total fracture risk. To our knowledge, this is the first meta-analysis of the relationship between serum vitamin D levels and fracture risk using age as an inclusion criterion. We researched the literature and browsed all relevant literature on total and hip fractures and we excluded all studies that did not meet the age requirements.

A few meta-analyses have been performed in this field, but they showed different results. Lv et al. [38] concluded that serum 25(OH)D levels were negatively correlated with hip fractures (RR (95% CI) 1.58 (1.41 to 1.77)). Feng et al. found a negative correlation to the risk of hip fracture as well, with total fracture in a meta-analysis of serum 25(OH)D and total and hip fracture risks. However, for total fractures, we have not concluded the correlation between serum 25(OH)D and total fractures in the elderly RR (95% Cl) 1.11 (0.99 to 1.24).

However, it was noteworthy that because of the different bone turnover rates, osteoporotic fractures in the elderly are divided into perimenopausal osteoporotic fractures and senile osteoporotic fractures. Evaluating these two types together adds uncertainty to the conclusion. Compared to the two meta-analyses above, our study focused on the population of elderly persons aged ≥ 60 years. These two factors may be the reasons for different conclusions.

Efficacy of vitamin D supplements in the elderly to prevent osteoporotic fractures is still uncertain. Heike et al. [39] conducted a meta-analysis of 12 RCTs to assess the relationship between oral vitamin D supplements and fracture risk in older adults, conclusion found that when oral vitamin D supplements reached a certain dose, the risk of fractures in the elderly might decrease. However, Jeffrey et al. [40] found that no dose of vitamin D was effective in preventing fractures in a meta-analysis of vitamin D supplementation and serum 25-hydroxyvitamin D levels and hip fractures. The reason of the discrepancy may be that the types of studies and the fracture site included in the two articles are different.

In recent years, serum markers and fracture risk have become a hot field with interest increasingly. Osteoporosis (OP) is a common and silent disease, and it is the primary cause of pathological fractures in the elderly [41]. The link between vitamin D deficiency and fractures has been proven in epidemiological studies [42]. However, there is no direct evidence linking serum vitamin D to fracture risk.

The inverse relationship between low serum 25(OH)D and fractures might have several plausible mechanisms. First, severe vitamin D deficiency causes rickets or osteomalacia, the consequences of vitamin D deficiency can cause osteoporosis and fractures, mineralization defects, which may lead to osteomalacia in the long term, and with muscle strength decreasing, falls and fractures will eventually occur. Second, vitamin D deficiency also causes higher secretion of parathyroid hormone (PTH) which leads to high bone turnover and increased bone resorption and eventually it will lead to bone loss or fracture. Hence, on the one hand, severe vitamin D deficiency causes a mineralization problem and osteomalacia and on the other hand increased PTH content can lead to increased bone conversion, bone resorption, and osteoporosis of bone. Third, several studies have shown a positive correlation between serum vitamin D and BMD [43–45]; low levels of serum vitamin D affect hip BMD. All mechanisms can cause fractures, especially hip fractures, in older people [46]. The previous meta-analysis did not reach a consistent conclusion about the relationship between serum vitamin D and fractures, which increased the uncertainty of the correlation between them.

The strengths of our meta-analysis are as follows: First, our study included a total of 41,738 participants and 5916 fracture events (including 3237 hip fractures), which greatly improved the statistical power of the analysis. Second, our evaluation criteria are based on observational studies. On the one hand, recall and selection bias in case-control studies can be reduced; on the other hand, it also provides additional data on the risk of fracture among participants. Third, all included studies were independently assessed by two reviewers. In case of discrepancies or disagreements pertaining to research inclusion and data interpretation, we resolved them through arbitration and discussion to reach a final agreement. Therefore, the errors in data were reduced. Fourth, all studies had a long period of follow-up and high scores in literature quality assessment (Newcastle-Ottawa scale).

However, the limitations of our study must be considered. First, despite the RR adjustment and the high-quality assessment scores of studies, our study is still influenced by a number of confounding factors that could be inherent in the obtained cohorts, which is a mutual disadvantage of all observational studies and meta-analyses, which can cause deviations in risk estimates. The serum vitamin D level is related to the sunlight duration. Due to the lack of data and the characteristics of prospective experiments, it is difficult to estimate the normal exposure time of participants, which may affect the final serum vitamin D test results. Second, differences in research methodology may be a source of heterogeneity. In the subgroup analysis of this study, we founded gender, location, the year that the study started, and analysis the sources of heterogeneity through these subgroups. Despite these factors that may reduce the strength of the conclusion, our forest plots indicate that relative risk is fairly consistent throughout the study.

## Conclusion

Our study indicates that compared to low serum 25(OH)D levels, high serum vitamin D protects against the risk of hip fracture in patients ≥ 60 years old with osteoporosis. However, serum 25(OH)D levels were not correlated to the total fracture risk. Although serum vitamin D levels cannot directly affect the risk of fractures, the indirect effects of low serum vitamin D levels on fractures suggest that vitamin D supplementation is still necessary.

## Data Availability

The datasets used and/or analyzed during the current study are available from the corresponding author on reasonable request.

## Abbreviations

25(OH)D: 25-Hydroxyvitamin D
BMD: Bone mineral density
BMI: Body mass index
CI: Confidence intervals
PA: Physical activity
PRISMA: Preferred Reporting Items for Systematic Reviews and Meta-analyses
RR: Relative risk
